# Do experiences with pregnancy, birth and postnatal care in Norway vary by the women’s geographic origin? a comparison of cross-sectional survey results

**DOI:** 10.1186/s12884-016-1214-3

**Published:** 2017-01-18

**Authors:** Ingeborg S. Sjetne, Hilde H. Iversen

**Affiliations:** The Knowledge Centre for the Health Services, Norwegian Institute of Public Health, PO Box 4404, Nydalen, N-0403 Oslo, Norway

## Abstract

**Background:**

A national survey was conducted to measure and benchmark women’s experiences with pregnancy, birth and postnatal care in Norway. The purpose of this secondary analysis is to explore potential variation in these experiences with regard to the survey respondents’ geographic origin.

**Methods:**

Data were collected in a national observational cross-sectional study, by a self-administered questionnaire and from registries. The questionnaire collects patient reported experience measures (PREMS) of mainly nontechnical aspects of the health-care services. While taking the clustered characteristics of the respondents into consideration, we compared the mean scores on 16 indexes between women of four different geographic origins using linear regression models.

**Results:**

The origin of the 4904 respondents were classified as Norway (*n* = 4028, 82%), Western Europe, North-America, Oceania (*n* = 233, 5%), Eastern Europe (*n* = 290, 6%), and Asia, Turkey, Africa, and South-America) (*n* = 353, 7%). The observed differences were moderate, and no consistency was present in the results in respect of direction or magnitude of the differences between the groups.

**Conclusions:**

With some important cautions, we conclude that this study did not detect systematic differences between groups of different geographic origin, in their experiences with pregnancy and maternity care in Norway.

## Background

Surveying patient-reported outcomes, including patient experiences, provides important information for the evaluation of health services. These surveys invite descriptions of mainly non-technical aspects of the health-care services and may involve different target populations, such as the general population, groups of service users, or patients with specific conditions. Many countries have programmes for assessing the quality of health care using surveys that describe the experiences of patients and other health-care users [1].

The information provided by the surveys is relevant to health policy-makers, health authorities, health-care managers at different levels, service providers, potential service users, and researchers. The explicit purpose of these surveys is fourfold: social legitimacy and control, business control, professional quality improvement, and to inform patient choice. Depending on the survey design, the results can be used to monitor health-system performance and/or inform quality improvement efforts at the level of service delivery.

Hospitals in Norway are obliged by law to have systems to collect user experiences with the services as a means to achieving user involvement. The responsibility for conducting health-care user experience surveys in Norway is lodged with the Knowledge Centre for the Health Services, recently merged with the Norwegian Institute of Public Health. The Knowledge Centre has developed a variety of data collection tools and surveyed a range of target groups. Data are collected through centralized and standardised routines, and can be aggregated on different organisational levels for comparisons and benchmarking. In order to have questionnaires with relevant contents and of an acceptable length, the topics explored should be meaningful to the majority of respondents.

In 2009, the Ministry of Health and Care Services issued a white paper entitled “A happy event. About a comprehensive pregnancy, birth and postnatal care” in which the Ministry commissioned a national user survey of women who had recently given birth and their partners [2]. The whole course of the health-care event (i.e. from pregnancy to postnatal care) was to be included, with special attention paid to immigrant women.

The extent of public responsibility for health care in Norway has been increasing since the beginning of the twentieth century, and today public sources account for over 85% of total health expenditure in Norway [3]. Strengthening the role of patients has been a policy priority since the turn of the millennium, manifested in a comprehensive Patients’ Rights Act. The object of this act is “*to help ensure that all citizens have equal access to good quality health care by granting patients’ rights in their relations with the health service”* [4]. Nonetheless, the patients’ relation to health services varies with characteristics like education, language skills, and health literacy. This implies that migrants may experience more impediments in their contacts with health care, compared with the general population. For women, the degree of self-determination in their social context also plays an important role [5].

The national survey was conducted in 2011, and included high quality complete data about the sampled women’s geographic origin. Both patient experience surveys and immigration are phenomena of international relevance, and the combination of the two is sparsely explored. We assume that this study therefore is interesting for service providers outside Norway.

## Objective

The purpose of this paper is to explore potential variation in experiences of maternity care in Norway with regard to the survey respondents’ geographic origin.

## Methods

The study is a secondary analysis of data from a national observational cross-sectional study, and data were collected by a self-administered questionnaire and from registries.

### Setting

The study object of the national survey was the entire care course from the first visit for pregnancy check-ups, through birth and postnatal care in birth institutions, and finally to the follow-up in public health clinics. In Norway, financing and delivery of prenatal monitoring and postnatal follow-up in health clinics are the responsibility of the municipality of residence of the individual women, while the birth institution—be it in a large hospital or a local maternity home—is the responsibility of the state, via specialized health care delivered by the hospital trusts.

### Eligibility and sampling

The point of care where the women were identified for potential inclusion was the institution where the birth took place. Women who gave birth in the last three months of 2011 in a Norwegian institution and who were 16 years old or older were eligible. In 2011, 99.2% of the 61321 births took place in an institution, the majority of the remaining 0.8% were unplanned home births [6]. Experiences from previous similar surveys led to a requirement of samples with 400 potential respondents from each hospital. The women were sampled randomly from institutions with more than 400 births during the inclusion period, and all women were included consecutively from institutions with fewer than 400 births. The Medical Birth Registry performed the sampling routine. Before any list of names and addresses was used for mailing, information from the National Registry (the national population register) was collected and any birth in which either the woman or child had died was excluded.

### Data collection

#### Questionnaire

The women responded to the survey using a pregnancy- and maternity-care patients’ experiences questionnaire (PreMaPEQ). The questionnaire collects patient reported experience measures (PREMS) and cover mainly nontechnical aspects of the health-care services, such as patient centeredness and patient information. The development and evaluation of the questionnaire is presented in detail elsewhere with the conclusion “*We conclude that the PreMaPEQ is a valid, reliable and acceptable instrument for collecting women’s experiences of the entire course of maternity care in health systems with features in common with the Norwegian health system*” [6, 7].

Most single items were scored on a 1–5 response format. Index scores represent the mean scores from the single items comprising the scale, transformed linearly to a 0–100 format (see Table 1). Index scores were based on items that were completed, and were calculated if at least half of the items in the index in question were completed.

**Table 1:** Questionnaire indexes

Care phases (Service level)	Indexes/Scales	No of items	Mean^a^	SD
Pregnancy (Municipal)				
	Check-ups by a general practitioner	5	*76.3*	*21.6*
	Check-ups by a midwife	5	*88.0*	*15.5*
	Ultrasound scan (performed in specialized health services)	2	*78.3*	*20.8*
	Information during pregnancy care	6	*66.6*	*21.1*
Birth (Specialized)				
	Personal relationships in the delivery ward	3	*81.0*	*18.7*
	Resources and organization in the delivery ward	7	*76.0*	*17.7*
	Attention to partner in the delivery ward	2	*84.2*	*18.0*
Postnatal stay (Specialized)				
	Personal relationships during your postnatal stay	3	*76.8*	*20.2*
	Resources and organization during your postnatal stay	6	*65.6*	*20.9*
	Attention to partner during your postnatal stay	2	*74.3*	*24.0*
	Information about women’s health during your postnatal stay	2	*58.3*	*26.2*
	Information and guidance about your child during your postnatal stay	4	*67.2*	*24.1*
Public health clinic (Municipal)				
	Personal relationships in the public health clinic	3	*76.8*	*20.2*
	Resources and organization in the public health clinic	3	*79.9*	*16.3*
	Information about women’s health in the public health clinic	2	*56.7*	*25.9*
	Information about your child in the public health clinic	4	*75.3*	*17.9*

Care phase, health service level and scale properties. *N* = 4904

^a^ Transformed linearly from a 1–5 scale to a 0–100 scale; high scores are favourable

The sampled women were contacted by mail about 17 weeks after the birth. They received a letter with information about the survey and an invitation to participate via the Internet. A specific username and password were included in the letter. Two reminders were sent to non-respondents, and both reminders included a printed questionnaire in addition to the username and password. The information letter and the internet version of the questionnaire was written in Norwegian and English. A printed English version of the questionnaire was sent by mail on request.

When all of the mailings were completed, the names and addresses were deleted from the data and the questionnaire answers were supplemented by clinical information from the Medical Birth Registry. Data on the geographic origin of the women included in the survey was collected from the National Registry via Statistics Norway, and added to the survey data.

Informed consent was considered expressed when, having received the written information, the women actively responded to the survey. The Regional Committee for Medical and Health Research Ethics (REK sør-øst D) approved the study.

### Variables

All of the 16 index scores derived from the questionnaire responses were used as outcome variables in the study, and the main explanatory variable is geographic origin.

In order to obtain categories with reasonably homogeneous groups and similar sizes, the women’s geographic origin was described by four categories used in previous studies of phenomena relevant to migration [8]: Norway; Western Europe, North-America, Oceania; Eastern Europe; and Asia, Turkey, Africa, and South-America.

### Analyses

Linear multiple regression models were used to assess the association between the index scores as dependent variables and geographic origin as independent variables.

The proportion of immigrants and people born in Norway to immigrant parents varies by geography. In 2011 it was highest in Oslo county (28.4%) and lowest in Nord-Trøndelag county (4.9%) [9]. Hence, the density of non-Norwegian users and personnel in health care varies between both hospitals and municipalities (Table 2). Before constructing the linear regression models, we assessed the potential need to control for the clustering effect caused by sampling the women via the institutions. With regard to the indexes describing municipal services, the women were sparsely distributed in a large number of municipalities with no relevant higher level clustering structure. The need for a multilevel approach in the analyses concerning experiences in the institutions was assessed by estimating the intra class correlation coefficient and the design effect [10].

**Table 2:** Distribution of women according to geographic origin. In percentages

		Norway	Western Europe, North America, Oceania	Eastern Europe	Asia, Turkey, Africa, South America
In counties					
	Min	74.2	1.0	2.6	1.4
	Max	91.4	7.9	10.1	11.6
In institutions					
	Min	70.5	0.8	2.4	1.1
	Max	97.4	12.8	15.4	15.5

The literature on patient experiences and satisfaction points to several individual characteristics that potentially influence the outcome measures, for example age, education, and employment [11–13]. We explored individual characteristics in the four groups of women and tested the differences with chi square statistics.

The women’s geographic origins were dummy coded and the effects on indexes describing municipal services were tested by ordinary least squares regression models. The effects of geographic origin on indexes describing experiences in the birth institutions was tested using multilevel regression models with random intercepts [14]. For all indexes the models were generated with and without individual characteristics that were associated with any of the indexes when tested with bivariate correlation (age, parity, self rated health, education, epidural/spinal anaesthesia in vaginal delivery, and caesarean section). We applied a significance level of 5% for the statistical tests.

The statistical software used was IBM SPSS Statistics for Windows, version 23 (IBM Corp, Armonk, N.Y., USA).

## Results

We contacted 8,670 eligible women, and 4,904 returned completed questionnaires, giving a 56.6% response rate. The proportion of older women, primipara and Western women was larger in the respondent group compared with the non-respondent group. More details are shown in a previous paper [7]. Among the respondents, 4,028 (82.1%) were born in Norway. The 876 women who were born outside of Norway came from 101 different countries, 233 (4.8%) were born in Western Europe, North America, or Oceania, 290 (5.9%) were born in Eastern Europe, and 353 (7.2%) were born in Asia, Turkey, Africa, or South America. Among Norway’s 429 municipalities in 2011, 396 were represented by 1–483 women in the survey data (mean = 12, median = 5).

The women gave birth in 51 different birth institutions, ten of them maternity homes. The maternity homes are small, local facilities that offer services to low risk women after strict selection criteria. We grouped 50 women from these facilities in one group, and hence had 42 institutions for the analyses with 26–269 respondents (mean = 117, median = 96).

For all the 9 indexes that describe experiences in specialized health care, the design effect was over 2 (mean 6.7), which is considered to indicate a need for a multi-level approach [10].

As seen in Table 3, the four groups of women differed in respect of both individual characteristics and characteristics of the institution where the birth took place. For example, the group from Eastern Europe had the largest proportion young women and the smallest proportion women with high parity. The group also had the highest proportion having epidural/spinal anaesthesia (excluding caesarean) and the lowest proportion caesarean sectios. The group from Asia, Turkey, Africa, and South America had the largest proportion women with primary school education and the smallest proportion women with education at university level. The largest proportion rating their health as Very good or Excellent was in the Norwegian group.

**Table 3:** Sample descriptives according to geographic origin (*N* = 4904)

	Norway		W Europe, N America, Oceania		Eastern Europe		Asia, Turkey, Africa, S America		Total		
	*N*	%	*N*	%	*N*	%	*N*	%	*N*	%	*p* for differences^a^
Age (years)											≤0.001
≤ 25	803	19.9	32	13.7	70	24.1	52	14.7	957	19.5	
> 25 ≤ 28	744	18.5	36	15.5	71	24.5	53	15.0	904	18.4	
> 28 ≤ 31	891	22.1	50	21.5	54	18.6	83	23.5	1078	22.0	
> 31 ≤ 35	950	23.6	53	22.7	64	22.1	94	26.6	1161	23.7	
> 35	640	15.9	62	26.6	31	10.7	71	20.1	804	16.4	
Parity											≤0.001
First	1894	47.0	109	46.8	177	61.0	164	46.5	2344	47.8	
Second	1367	33.9	89	38.2	88	30.3	118	33.4	1662	33.9	
Third	579	14.4	24	10.3	22	7.6	45	12.7	670	13.7	
Fourth or more	188	4.7	11	4.7	3	1.0	26	7.4	228	4.6	
Birth											
Single birth	3972	98.6	226	97.0	287	99.0	350	99.2	4835	98.6	0.146
Epidural or spinal anaesthesia (excluding caesarean)	1058	26.3	62	26.6	102	35.2	102	28.9	1324	27.0	0.009
Mode of delivery											
Emergency caesarean	451	11.2	32	13.7	27	9.3	61	17.3	571	11.6	0.003
Planned caesarean	246	6.1	14	6.0	14	4.8	20	5.7	294	6.0	0.835
Regional health authority											0.012
Southeast	2121	52.7	146	62.7	164	56.6	208	58.9	2639	53.8	
West	755	18.7	30	12.9	49	16.9	67	19.0	901	18.4	
Central	652	16.2	29	12.4	50	17.2	50	14.2	781	15.9	
North	500	12.4	28	12.0	27	9.3	28	7.9	583	11.9	
Institution size (no. of births per year)											0.009
< 49 + other/unspecified	34	0.8	0	0	5	1.7	2	0.6	41	0.8	
50–499	586	14.5	32	13.7	46	15.9	37	10.5	701	14.3	
500–1499	1003	24.9	45	19.3	77	26.6	69	19.5	1194	24.3	
1500–2999	1336	33.2	100	42.9	95	32.8	121	34.3	1652	33.7	
≤ 3000	1069	26.5	56	24.0	67	23.1	124	35.1	1316	26.8	
Municipality size (no. of inhabitants)											0.000
< 5000	538	13.4	24	10.3	38	13.1	33	9.3	633	12.9	
5000-9999	576	14.3	29	12.4	45	15.5	33	9.3	683	13.9	
10000-19999	682	16.9	35	15.0	45	15.5	43	12.2	805	16.4	
20000-49999	985	24.5	59	25.3	70	24.1	85	24.1	1199	24.4	
> 50000	1247	31.0	86	36.9	92	31.7	159	45.0	1584	32.3	
Marital status^b^											0.727
Neither married nor cohabiting	125	3.1	6	2.6	6	2.1	12	3.4	149	3.0	
Education^b^											0.000
Primary school	129	3.3	6	2.6	8	3.0	62	18.8	205	4.3	
Secondary school	1173	29.6	54	23.4	70	26.2	96	29.1	1393	29.1	
University undergraduate	1571	39.7	82	35.5	92	34.5	119	36.1	1864	38.9	
University postgraduate	1088	27.5	89	38.5	97	36.3	53	16.1	1327	27.7	
Main activity when not on maternity leave^b^											0.000
Working	3322	83.8	188	81.4	162	60.7	167	50.6	3839	80.1	
Sick leave or welfare allowances	117	3.0	4	1.7	3	1.1	5	1.5	129	2.7	
Education	272	6.9	13	5.6	8	3.0	57	17.3	350	7.3	
Homemaking	43	1.1	4	1.7	30	11.2	49	14.8	126	2.6	
Unemployed	176	4.4	14	6.1	48	18.0	41	12.4	279	5.8	
Other	35	0.9	8	3.5	16	6.0	11	3.3	70	1.5	
Self-rated health^b^											0.000
Poor	41	1.0	2	0.9	3	1.1	6	1.8	52	1.1	
Fair	223	5.6	20	8.7	19	7.1	50	15.2	312	6.5	
Good	1058	26.7	63	27.3	132	49.4	113	34.2	1366	28.6	
Very good	1780	45.0	86	37.2	82	30.7	105	31.8	2053	42.9	
Excellent	854	21.6	60	26.0	31	11.6	56	17.0	1001	20.9	

^a^:*χ* ^2^ test

^b^:Data from questionnaire

We built two regression models each for municipal services and specialized services. In the first, unadjusted model, dummy codes for geographic origin was the only explanatory variable. This would show the effect of geographic origin. In the second model we added a set of individual characteristics (age, parity, self rated health, education, epidural/spinal anaesthesia in vaginal delivery, and caesarean sectio) that were associated with any of the outcome variables when tested with bivariate correlations. The latter, adjusted model would show the effect of geographic origin after correction for uneven distributions of other influences on the outcome variables in the different groups.

Table 4 presents the results for services provided in municipal health care; that is during pregnancy and after the hospital stay. There are differences on all the indexes in the unadjusted model. Compared to women from Norway, the women from Western Europe, North-America, Oceania had given lower scores on five of the seven indexes, the women from Eastern Europe had given lower scores on three indexes, and the women from Asia, Turkey, Africa, South-America had given lower scores on two and higher scores on three indexes. The largest differences is found in the indexes measuring experiences with information. Experiences with information during pregnancy care, about women’s health and about the child in the public health clinic were all described poorer by the women from Western Europe, North-America, Oceania, and better by the women from Asia, Turkey, Africa, South-America, compared with the Norwegian women. Adding individual characteristics to the explanatory variables reduced the total number of negative differences from ten to five and increased the number of positive differences from three to six.

**Table 4:** Effect of geographic origin on the mean scores for municipal services

	Unadjusted					Adjusted^a^				
				95% CI					95% CI	
	Estimate	Std. error	Sig.	Lower bound	Upper bound	Estimate	Std. error	Sig.	Lower bound	Upper bound
Check-up by a general practitioner										
Norway (reference value)	-	-	-	-	-	-	-	-	-	-
Western Europe, North-America, Oceania	−4.81	1.65	.004	−8.04	−1.58	−4.88	1.63	.003	−8.09	−1.68
Eastern Europe	0.09	1.55	.955	−2.95	3.13	2.04	1.55	.188	−1.00	5.08
Asia, Turkey, Africa, South-America	0.84	1.40	.547	−1.91	3.60	1.12	1.41	.427	−1.65	3.89
Check-up by a midwife										
Norway (reference value)	-	-	-	-	-	-	-	-	-	-
Western Europe, North-America, Oceania	−2.34	1.11	.036	−4.52	−0.16	−2.32	1.11	.037	−4.49	−0.14
Eastern Europe	−4.10	1.08	.000	−6.22	−1.99	−3.44	1.08	.001	−5.56	−1.32
Asia, Turkey, Africa, South-America	−4.61	0.97	.000	−6.51	−2.71	−4.11	0.98	.000	−6.03	−2.20
Information during pregnancy care										
Norway (reference value)	-	-	-	-	-	-	-	-	-	-
Western Europe, North-America, Oceania	−5.73	1.46	.000	−8.59	−2.87	−5.38	1.45	.000	−8.23	−2.54
Eastern Europe	1.59	1.36	.243	−1.08	4.26	3.05	1.37	.025	0.38	5.73
Asia, Turkey, Africa, South-America	2.72	1.22	.026	0.32	5.12	2.66	1.23	.031	0.25	5.08
Personal relationships in the public health clinic										
Norway (reference value)	-	-	-	-	-	-	-	-	-	-
Western Europe, North-America, Oceania	−0.75	1.07	.483	−2.84	1.34	−0.61	1.06	.568	−2.69	1.48
Eastern Europe	−2.61	1.01	.010	−4.59	−0.63	−1.83	1.02	.072	−3.82	0.16
Asia, Turkey, Africa, South-America	−2.24	0.91	.014	−4.03	−0.45	−1.64	0.92	.075	−3.45	0.17
Information about women’s health in the public health clinic										
Norway (reference value)	-	-	-	-	-	-	-	-	-	-
Western Europe, North-America, Oceania	−3.56	1.77	.044	−7.03	−0.09	−3.01	1.76	.087	−6.47	0.44
Eastern Europe	2.62	1.67	.118	−0.66	5.90	4.56	1.68	.007	1.27	7.85
Asia, Turkey, Africa, South-America	4.96	1.51	.001	2.01	7.92	5.35	1.52	.000	2.37	8.33
Information about your child in the public health clinic										
Norway (reference value)	-	-	-	-	-	-	-	-	-	-
Western Europe, North-America, Oceania	−2.40	1.22	.049	−4.79	−0.01	−1.75	1.21	.148	−4.12	0.62
Eastern Europe	1.82	1.15	.113	−0.43	4.08	3.23	1.15	.005	0.97	5.49
Asia, Turkey, Africa, South-America	4.73	1.04	.000	2.69	6.76	4.57	1.04	.000	2.53	6.61
Resources and organisation in the public health clinic										
Norway (reference value)	-	-	-	-	-	-	-	-	-	-
Western Europe, North-America, Oceania	−0.95	1.11	.391	−3.13	1.22	−0.73	1.11	.509	−2.90	1.44
Eastern Europe	−2.54	1.05	.015	−4.60	−0.49	−1.57	1.05	.136	−3.64	0.49
Asia, Turkey, Africa, South-America	−0.69	0.95	.465	−2.56	1.17	−0.45	0.96	.642	−2.33	1.44

Scale 0–100; high scores are favourable. Multiple linear regression

^a^: Adjusted for age, parity, self rated health, education, epidural/spinal anaesthesia in vaginal delivery, and caesarean section

Table 5 presents the results for services provided by institutions in the specialized health care. There are statistically significant differences on all but one index in the partly adjusted model. Compared to women from Norway, the women from Western Europe, North-America, Oceania had given lower scores on two of the nine indexes, the women from Eastern Europe had given lower scores on one and higher scores on six indexes, and the women from Asia, Turkey, Africa, South-America had given lower scores on two and higher scores on three indexes. The index describing information and guidance about your child during postnatal stay shows the largest difference. The consequence of adding individual characteristics to the explanatory variables in the model was a reduction in the total number of negative differences from five to four. The estimated differences between the groups increased and the p-values decreased.

**Table 5:** Effect of geographic origin on the mean scores for specialized care services

			Partly adjusted^a^					Adjusted^b^				
						95% CI					95% CI	
	ICC	Design effect	Estimate	Std. error	Sig.	Lower bound	Upper bound	Estimate	Std. error	Sig.	Lower bound	Upper bound
Ultrasound scan	0.02	3.17										
Norway (reference value)			-	-	-	-	-	-	-	-	-	-
Western Europe, North-America, Oceania			−1.61	1.41	.255	−4.38	1.16	−1.83	1.40	.193	−4.58	0.93
Eastern Europe			−4.76	1.32	.000	−7.35	−2.17	−2.51	1.34	.062	−5.15	0.13
Asia, Turkey, Africa, South-America			−7.37	1.20	.000	−9.72	−5.03	−6.51	1.22	.000	−8.89	−4.12
Personal relationships in the delivery ward	0.03	4.55										
Norway (reference value)			-	-	-	-	-	-	-	-	-	-
Western Europe, North-America, Oceania			−1.76	1.32	.185	−4.35	0.84	−1.89	1.30	.147	−4.45	0.66
Eastern Europe			−0.72	1.21	.554	−3.10	1.66	1.23	1.22	.316	−1.17	3.62
Asia, Turkey, Africa, South-America			−0.60	1.13	.598	−2.81	1.62	0.54	1.15	.637	−1.70	2.79
Attention to partner in the delivery ward	0.04	5.74										
Norway (reference value)			-	-	-	-	-	-	-	-	-	-
Western Europe, North-America, Oceania			−0.50	1.28	.698	−3.01	2.02	−0.50	1.28	.693	−3.01	2.00
Eastern Europe			2.59	1.18	.028	0.27	4.90	3.63	1.19	.002	1.29	5.97
Asia, Turkey, Africa, South-America			−4.18	1.09	.000	−6.31	−2.04	−3.40	1.11	.002	−5.58	−1.22
Resources and organization in the delivery ward	0.04	5.55										
Norway (reference value)			-	-	-	-	-	-	-	-	-	-
Western Europe, North-America, Oceania			−2.82	1.25	.023	−5.26	−0.38	−3.01	1.22	.014	−5.40	−0.62
Eastern Europe			−0.59	1.15	.607	−2.83	1.66	1.21	1.15	.290	−1.03	3.46
Asia, Turkey, Africa, South-America			0.51	1.06	.632	−1.57	2.59	1.56	1.07	.146	−0.54	3.65
Personal relationships during your postnatal stay	0.06	8.02										
Norway (reference value)			-	-	-	-	-	-	-	-	-	-
Western Europe, North-America, Oceania			−0.23	1.37	.864	−2.92	2.45	−0.70	1.36	.607	−3.36	1.96
Eastern Europe			3.36	1.31	.010	0.79	5.92	4.93	1.31	.000	2.37	7.50
Asia, Turkey, Africa, South-America			0.61	1.18	.603	−1.69	2.92	1.63	1.19	.169	−0.69	3.96
Information about women’s health during your postnatal stay	0.03	4.67										
Norway (reference value)			-	-	-	-	-	-	-	-	-	-
Western Europe, North-America, Oceania			−1.85	1.80	.304	−5.37	1.67	−2.07	1.79	.248	−5.58	1.44
Eastern Europe			5.36	1.72	.002	1.98	8.74	7.82	1.74	.000	4.41	11.22
Asia, Turkey, Africa, South-America			4.25	1.54	.006	1.24	7.26	4.84	1.56	.002	1.78	7.90
Information and guidance about your child during your postnatal stay	0.04	5.60										
Norway (reference value)			-	-	-	-	-	-	-	-	-	-
Western Europe, North-America, Oceania			−4.12	1.66	.013	−7.37	−0.88	−4.38	1.65	.008	−7.61	−1.14
Eastern Europe			7.30	1.57	.000	4.21	10.38	8.23	1.58	.000	5.12	11.33
Asia, Turkey, Africa, South-America			3.20	1.41	.023	0.43	5.97	4.09	1.43	.004	1.28	6.90
Attention to partner during your postnatal stay	0.10	12.44										
Norway (reference value)			-	-	-	-	-	-	-	-	-	-
Western Europe, North-America, Oceania			−0.85	1.61	.597	−4.00	2.30	−1.27	1.60	.425	−4.40	1.86
Eastern Europe			6.53	1.54	.000	3.52	9.55	7.65	1.54	.000	4.63	10.68
Asia, Turkey, Africa, South-America			−1.71	1.38	.215	−4.42	1.00	−1.32	1.40	.345	−4.07	1.42
Resources and organization during your postnatal stay	0.08	10.30										
Norway (reference value)			-	-	-	-	-	-	-	-	-	-
Western Europe, North-America, Oceania			−0.45	1.40	.746	−3.19	2.29	−0.92	1.38	.506	−3.62	1.79
Eastern Europe			5.96	1.34	.000	3.34	8.59	8.03	1.33	.000	5.41	10.64
Asia, Turkey, Africa, South-America			5.05	1.20	.000	2.70	7.41	5.63	1.21	.000	3.26	7.99

Scale 0–100; high scores are favourable. Two-level multiple linear regression

^a^: Adjusted for clustering within institutions

^b^: Adjusted for clustering within institutions and individual characteristics (age, parity, self rated health, education, epidural/spinal anaesthesia in vaginal delivery, caesarean section)

The scores from women from Eastern Europe and Asia, Turkey, Africa, South-America are higher compared to the Norwegian women’s scores on all the five indexes that describe experiences with information in the fully adjusted models. On two of these indexes the scores from women from Western Europe, North-America, Oceania are lower in the same comparison.

The overall impression of the results is that the experiences with maternity services vary to some extent by geographic origin. There is no consistency in respect of the direction and magnitude of the intergroup differences.

## Discussion

With the objective of studying potential variation according to geographic origin in experiences of maternity care in Norway, we found differences in all but one indexes in this study. The differences were moderate, and no consistency was present in the results in respect of direction or magnitude of the differences between the groups.

The tendency of women from Eastern Europe and Asia, Turkey, Africa, South-America to describe the experiences with information more positive in comparison to Norwegian women is the most consistent finding. We can only speculate about what this indicates. This may indicate for example that the health care personnel pay more attention to meeting these groups’ need for information or that the groups are less informed to begin with, and hence find the information provided more useful.

The way the women were classified is a possible limitation, as the purpose is to compare homogeneous subgroups. The present study uses geographic origin as a proxy for ethnicity, but the resulting groups most likely still have much internal heterogeneity, as shown in Table 3. There is no tradition in Norway for collecting data pertaining to ethnicity, and previous international research has shown that the quality of such data may be weak [15]. However, in delivering health services to a population of increasing geographical mobility, data pertaining to ethnicity or origin is important information that should not be neglected, even if considered sensitive. In this study, all information about geographic origin was collected for the whole sample from the same source, the national population register. The register includes all legal residents in Norway and the quality is considered very good for statistical purposes [16]. This is an important feature, which contributes to the validity and reliability of the results.

The questionnaire was translated into English as the only alternative to Norwegian. It is possible that translating into more foreign languages would have facilitated the participation of immigrant women with weaker language skills, and that this in turn would have impacted the findings. However, there would still be the problem of distributing the letters and questionnaires in accordance with the respondent’s language preferences and skills. Further translations were not done due to their high cost and limited expected benefit.

In order to limit the response burden and cost, it was a strict criterion in the questionnaire development process that all questions should be of relevance to the majority of the respondents. Hence, there were no questions tapping directly into possible experiences of disparity in the services. This implies that data collection using a self-administered questionnaire is unsuitable for capturing more particular information, or information that is relevant for only a minority in the sample. It is possible that the results would have been different, had items of this kind been developed and included.

We cannot exclude that the study results are influenced by response bias differences between the groups [17, 18]. For example, the degree of social desirability bias may be associated with the established level of trust between the respondent and public authorities in general.

As health-care user surveys are becoming a mainstream tradition in Norway, Norwegian women may be more familiar with this mode of communication. The women who are least capable of sharing their responses may also be among the most vulnerable, and their specific situation cannot be captured by the method in use. We cannot claim that the study results are representative for women in this subgroup. Lacking language skills is probably an important obstacle leading to different response rates in the groups. Frequent changes of residence among newly arrived immigrants may make the mail distribution fallible.

There are indications that there are disparities that are neglected by the method used in the present study. It has been found in qualitative studies that there are subgroups among immigrant women who are vulnerable and that the flexibility of maternity services should be improved in order to better meet their specific needs [19, 20]. A recent Norwegian registry study showed that, among the compared groups, the risk of adverse obstetric outcomes was higher among women of African and Asian descent [21]. The present national survey was customized to measure experiences at the group level. To study particularities in women’s backgrounds (e.g. minorities) or birth experiences (e.g. home or transport deliveries) calls for more individual and flexible approaches.

Despite the stated reservations and methodological considerations, the results from the current study represent an important start in exploring potential variation in experiences of maternity care in Norway with regard to the respondents’ geographic origin. This knowledge is potentially relevant when monitoring health-system performance as well as quality improvement efforts, but future surveys as well as research should address the potential challenges addressed above.

## Conclusion

With the important reservations presented above, we conclude that this study did not detect systematic differences between groups of different geographic origin, in their experiences with maternity care in Norway.
